# Kessler-10 Psychological Distress Score Is Independently Associated with SYNTAX I and SYNTAX II Scores in Patients with Acute Coronary Syndrome

**DOI:** 10.3390/medicina62081440

**Published:** 2026-07-24

**Authors:** Fikret Keles, Alp Yildirim, Ahmet Ridvan Bilgic, Muzeyyen Gizem Parmak, Alperen Tas, Mustafa Celik, Muhammet Salih Ates, Erdogan Sokmen

**Affiliations:** 1 Department of Cardiology, Kirsehir Ahi Evran University Faculty of Medicine, Kirsehir 40100, Türkiye; dr.alp.yildirim.1907@gmail.com (A.Y.); mgizemp@gmail.com (M.G.P.); m.salih.ates@gmail.com (M.S.A.); erdoganmen@gmail.com (E.S.); 2 Department of Chest Diseases, Kirsehir Training and Research Hospital, Kirsehir 40100, Türkiye; dr.ridvanbilgic@gmail.com; 3 Department of Cardiology, Kirsehir Training and Research Hospital, Kirsehir 40100, Türkiye; alperentas555@hotmail.com; 4 Department of Cardiology, Necmettin Erbakan University Meram Faculty of Medicine, Konya 42090, Türkiye; muscelik50@gmail.com

**Keywords:** Kessler-10, psychological distress, psychocardiology, acute coronary syndrome, SYNTAX score, SYNTAX score II, coronary artery disease

## Abstract

*Background and Objectives*: Psychological distress is common in acute coronary syndrome (ACS), yet its relationship with objective coronary anatomical complexity is incompletely understood. The Kessler Psychological Distress Scale-10 (K10) is a brief self-report instrument that measures non-specific psychological distress over the preceding four weeks. We investigated whether the K10 score is associated with the anatomical SYNTAX I score and the clinical–anatomical SYNTAX II PCI score in patients hospitalized with ACS. *Materials and Methods*: This prospective, single tertiary-center observational study assessed 865 adult patients with suspected or confirmed ACS between 6 January 2026 and 5 June 2026. After applying predefined inclusion and exclusion criteria, 750 consecutive eligible patients with complete K10, SYNTAX I, and SYNTAX II PCI data were included in the final analytic cohort. The cohort consisted of STEMI in 337 patients (44.9%), NSTEMI in 310 (41.3%), and unstable angina pectoris in 103 (13.7%). The validated Turkish version of the K10 scale was administered after pain control and hemodynamic stabilization, within 24–48 h of admission, by trained study personnel blinded to the final SYNTAX analysis. SYNTAX I and SYNTAX II PCI scores were calculated by two blinded interventional cardiologists. The primary endpoint was SYNTAX I score ≥ 23; the key secondary endpoint was SYNTAX II PCI score ≥ 36, interpreted as a cohort-based high clinical–anatomical risk threshold. *Results*: Mean age was 61.3 ± 11.6 years, and 543 patients (72.4%) were men. Mean K10, SYNTAX I, and SYNTAX II PCI scores were 23.7 ± 6.7, 22.4 ± 9.5, and 31.2 ± 9.6, respectively. K10 score correlated with SYNTAX I (Spearman rho = 0.417, *p* < 0.001) and SYNTAX II PCI (rho = 0.348, *p* < 0.001). Each 5-point increase in K10 was independently associated with SYNTAX I ≥ 23 (extended model-adjusted odds ratio [OR] 1.84, 95% confidence interval [CI] 1.59–2.12; *p* < 0.001) and SYNTAX II PCI ≥ 36 (extended model-adjusted OR 2.26, 95% CI 1.85–2.75; *p* < 0.001). K10 predicted SYNTAX I ≥ 23 with an area under the curve (AUC) of 0.706 (95% CI 0.674–0.744; cutoff ≥ 24) and SYNTAX II PCI ≥ 36 with an AUC of 0.693 (95% CI 0.654–0.736; cutoff ≥ 26). Adding K10 to the extended clinical model improved cross-validated AUC for SYNTAX I ≥ 23 from 0.708 to 0.774 and for SYNTAX II PCI ≥ 36 from 0.871 to 0.905; bootstrap optimism-corrected AUCs were 0.754 and 0.899, respectively. The findings remained consistent in sensitivity analyses excluding prior CABG patients, adjusting for psychiatric history/medication use, and evaluating patients whose K10 was completed before angiography. *Conclusions*: In this prospective ACS cohort, early in-hospital assessment of K10-defined recent psychological distress was independently associated with both anatomical and clinical–anatomical coronary complexity. K10 should not replace angiographic risk scoring, but it may help identify a psychocardiological phenotype characterized by higher coronary disease burden and greater clinical vulnerability.

## 1. Introduction

Acute coronary syndrome (ACS) is a heterogeneous clinical spectrum that includes ST-segment elevation myocardial infarction (STEMI), non-ST-segment elevation myocardial infarction (NSTEMI), and unstable angina pectoris (USAP). The 2023 European Society of Cardiology guideline emphasizes that patients presenting with ACS require early diagnosis, risk stratification, invasive assessment when appropriate, revascularization, and long-term secondary prevention within an integrated pathway [1,2,3]. Although the immediate focus in ACS is coronary reperfusion and antithrombotic therapy, the event also represents a severe psychological stressor. Fear of death, chest pain, loss of control, insomnia, anticipatory anxiety, and depressive symptoms are common during and after hospitalization. In routine cardiology practice, however, psychological distress is often regarded as a parallel problem rather than as a variable that may be related to cardiovascular disease burden.

The SYNTAX I score is an angiographic scoring system that grades the anatomical complexity of coronary artery disease according to lesion location, number, bifurcation or trifurcation anatomy, chronic total occlusion, calcification, tortuosity, thrombus, and diffuse disease in vessels of clinically relevant diameter [4,5]. It has been used both in stable coronary disease and ACS to stratify patients into low, intermediate, and high anatomical complexity groups [6]. SYNTAX II was later developed by Farooq et al. [7] to integrate the anatomical SYNTAX score with clinical variables such as age, sex, creatinine clearance, left ventricular ejection fraction, chronic obstructive pulmonary disease, peripheral vascular disease, and unprotected left main disease. This clinical–anatomical approach provides more individualized risk estimation than anatomical scoring alone.

The Kessler Psychological Distress Scale-10 (K10) was developed by Kessler et al. [8] as a short screening scale for non-specific psychological distress. The instrument captures symptoms related to anxiety and depression during the preceding four weeks, is easy to administer, and has been used in epidemiological, primary care, emergency, addiction, oncology, and general medical settings [9,10]. Andrews and Slade [11] provided a widely cited interpretive framework for K10 categories, while later clinical guidance emphasized that the tool is a screening measure rather than a diagnostic psychiatric interview. The Turkish adaptation by Altun et al. [12] showed excellent internal consistency, test–retest reliability, and ROC performance, supporting the use of the validated Turkish version of the K10 scale in clinical research.

Beyond its psychometric use, K10-defined psychological distress has been associated with incident cardiovascular disease and adverse cardiovascular outcomes in population-based and clinical cohorts [13,14,15,16]. These studies support the broader cardiometabolic relevance of psychological distress, but most did not evaluate objective angiographic complexity. Therefore, linking K10 with SYNTAX-based anatomical and clinical–anatomical scores may provide a more direct bridge between psychocardiological vulnerability and coronary disease burden.

The relationship between psychological distress and coronary disease complexity is biologically plausible. Distress may be associated with sympathetic activation, hypothalamic–pituitary–adrenal axis dysregulation, endothelial dysfunction, inflammatory activation, platelet reactivity, adverse sleep patterns, smoking, reduced physical activity, poor medication adherence, and delayed healthcare seeking [17,18,19,20,21]. Conversely, severe and diffuse coronary artery disease may itself produce more intense symptoms, more frightening hospitalization experiences, and higher distress. Therefore, the association may be bidirectional, and a well-designed ACS study must carefully define the timing of psychological assessment, stabilization criteria, and blinding of angiographic analysis.

Only a limited number of studies have directly linked psychological scales with objective coronary complexity. Kaya et al. [22] reported that Hospital Anxiety and Depression Scale scores were associated with SYNTAX scores in patients with ACS undergoing percutaneous coronary intervention. Ekici et al. [23] also described a relationship between emotional status, health-related quality of life, and coronary artery disease severity among patients undergoing coronary angiography. However, evidence remains limited, sample sizes have generally been modest, and K10 has not been specifically evaluated in relation to both SYNTAX I and SYNTAX II in a large ACS cohort.

In this study, we aimed to investigate the relationship between K10 score and both SYNTAX I and SYNTAX II PCI scores in patients hospitalized with ACS. We hypothesized that higher K10-defined psychological distress would be independently associated with higher anatomical and clinical–anatomical coronary complexity, and that adding K10 to clinical variables would improve discrimination for higher SYNTAX-based risk categories.

## 2. Materials and Methods

### 2.1. Study Design and Setting

This was a prospective, single tertiary-center, observational cohort study including consecutive adult patients admitted with ACS and referred for invasive coronary angiography. A prospective design was methodologically appropriate because K10 is a patient-reported measure with a fixed four-week reference period and requires standardized administration conditions; retrospective chart extraction would not reliably capture psychological distress at a comparable phase of ACS hospitalization. Patients were assessed for eligibility from 6 January 2026 to 5 June 2026 at the Department of Cardiology, Kirsehir Ahi Evran University Faculty of Medicine, a high-volume tertiary cardiology center with 24 h primary PCI capability. As the sole tertiary referral center for the entire province and surrounding rural districts, all suspected or confirmed ACS cases in the region are directly transferred to our institution, enabling the rapid enrollment of a large consecutive cohort. The study flow was structured to separate clinical stabilization, K10 administration, angiographic analysis, and SYNTAX scoring as much as possible. Exposure assessment was performed before the research SYNTAX results were available to the K10 assessor and before K10 scores were available to the angiographic readers. We used the STROBE reporting guideline to draft this manuscript [24].

The study was conducted as an explanatory risk-stratification study rather than as a diagnostic psychiatric validation study. Therefore, the exposure variable was the K10 score, analyzed primarily as a continuous variable and secondarily as clinically interpretable distress categories. The outcomes were SYNTAX I and SYNTAX II PCI scores, analyzed both as continuous scores and as prespecified higher-complexity binary endpoints. Reporting followed STROBE-oriented observational study principles, with transparent descriptions of eligibility criteria, timing of questionnaire administration, missing data handling, confounder selection, model building, discrimination, calibration, internal validation, and sensitivity analyses.

### 2.2. Study Size and Patient Flow

**Figure 1:**
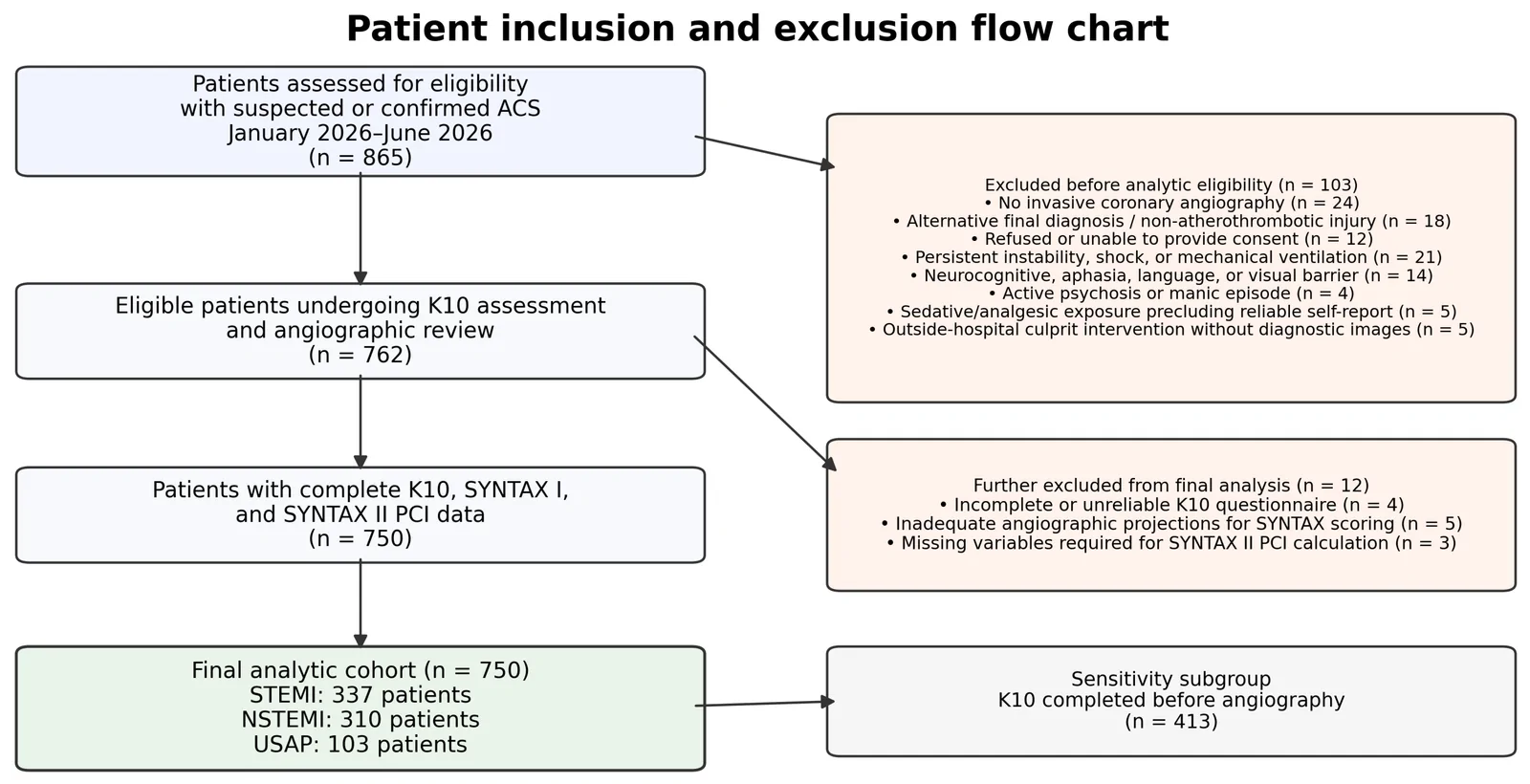
Patient inclusion and exclusion flow chart. Among 865 patients assessed for eligibility with suspected or confirmed ACS between 6 January 2026 and 5 June 2026, 750 consecutive eligible patients with complete K10, SYNTAX I, and SYNTAX II PCI data were included in the final analytic cohort. Patients with previous CABG were not excluded when native-vessel SYNTAX assessment was feasible; this subgroup was evaluated in prespecified sensitivity analyses. ACS, acute coronary syndrome; CABG, coronary artery bypass grafting; K10, Kessler Psychological Distress Scale-10; PCI, percutaneous coronary intervention; SYNTAX, Synergy Between PCI With Taxus and Cardiac Surgery.

No formal pre-study power calculation was used to determine the final cohort size. During the recruitment period from 6 January 2026 to 5 June 2026, 865 patients with suspected or confirmed ACS were assessed for eligibility. After applying predefined inclusion and exclusion criteria, 750 consecutive eligible patients with complete K10, SYNTAX I, and SYNTAX II PCI data were included in the final analytic cohort. This final sample provided adequate precision for multivariable modeling, with 387 events for SYNTAX I ≥23 and 220 events for SYNTAX II PCI ≥36, allowing adjustment for clinically relevant covariates while maintaining an acceptable events-per-variable ratio. The patient selection process is summarized in Figure 1.

### 2.3. Patient Inclusion and Exclusion Criteria

Patients were eligible for inclusion if they were aged 18 years or older, were hospitalized with a final diagnosis of STEMI, NSTEMI, or USAP, underwent invasive coronary angiography during the index hospitalization, had angiographic images suitable for native-vessel SYNTAX I scoring, had the clinical variables required for SYNTAX II PCI calculation, were able to complete the validated Turkish version of the K10 scale after pain control and hemodynamic stabilization, and provided written informed consent. ACS diagnosis was based on symptoms, electrocardiographic findings, high-sensitivity cardiac troponin results, and angiographic/clinical evaluation in accordance with contemporary guideline definitions [1]. STEMI was defined by ischemic symptoms with persistent ST-segment elevation or new left bundle branch block equivalent requiring urgent reperfusion. NSTEMI was defined by ischemic symptoms with elevated cardiac troponin without persistent ST-segment elevation. USAP was defined by ischemic symptoms at rest or crescendo angina without biomarker evidence of myocardial necrosis. The Global Registry of Acute Coronary Events (GRACE) risk score was also calculated for conventional risk stratification [25,26].

Patients were excluded if they had an alternative final diagnosis rather than ACS, including myocarditis, Takotsubo syndrome, pulmonary embolism, aortic dissection, or non-ischemic myocardial injury; type 2 myocardial infarction due to non-coronary supply-demand imbalance without culprit atherosclerotic coronary disease; no invasive coronary angiography during the index hospitalization; refusal or inability to provide informed consent; cardiogenic shock or persistent hemodynamic instability at the time planned for K10 assessment; mechanical ventilation; active delirium, severe cognitive impairment, severe aphasia, known dementia, or severe visual/language barrier preventing questionnaire completion; active psychosis or manic episode; sedative or analgesic exposure precluding reliable self-report; culprit lesion treated at an outside hospital before transfer without available diagnostic angiographic images; incomplete or unreliable K10 questionnaire; inadequate angiographic projections for SYNTAX scoring; or missing variables required for SYNTAX II PCI calculation. Previous coronary artery bypass grafting was not considered an automatic exclusion criterion. Patients with prior CABG were included when the native coronary tree could be evaluated adequately for SYNTAX scoring; because SYNTAX assessment after CABG is less standardized, all main analyses were repeated after excluding these patients as a prespecified sensitivity analysis.

### 2.4. Timing and Administration of the K10 Questionnaire

The validated Turkish version of the Kessler Psychological Distress Scale-10 (K10) [12] was administered by a trained research nurse or physician who was not involved in SYNTAX scoring. The K10 was selected over other instruments (such as PHQ-9 or HADS) because it is a rapid, 10-item global screener that efficiently captures both anxiety and depressive symptoms simultaneously, making it highly practical for a fast-paced acute cardiovascular setting. The questionnaire was completed in a quiet coronary care unit or cardiology ward room after pain control and hemodynamic stabilization. Stabilization was operationally defined as the absence of ongoing severe ischemic chest pain, systolic blood pressure ≥ 90 mmHg without escalating vasopressor support, no need for invasive ventilation, no active delirium, and clinical ability to answer self-report questions. In STEMI patients, K10 was never administered before reperfusion and was completed after primary PCI, usually within 24–48 h of admission. In NSTEMI and USAP patients, K10 was completed after initial stabilization and before angiography whenever clinically feasible; if angiography had already been performed, the questionnaire was administered before the formal research SYNTAX result was known to the patient and to the K10 assessor. The exact interval from admission to K10 completion and the timing of K10 relative to angiography were recorded. These variables were used in sensitivity analyses to address the possibility that knowledge of angiographic severity or the acute procedural experience could influence patient-reported distress.

The K10 consists of 10 items assessing how often the patient experienced symptoms such as tiredness for no good reason, nervousness, uncontrollable nervousness, hopelessness, restlessness, inability to sit still, depressed mood, effortfulness, sadness, and worthlessness in the past four weeks. Each item is scored from 1 to 5, corresponding to none of the time, a little of the time, some of the time, most of the time, and all the time. Total score ranges from 10 to 50, with higher scores indicating greater psychological distress [8,11]. Questions 3 and 6 follow the conventional skip logic: if the preceding screening item is answered as none of the time, the dependent item automatically receives a score of 1 [11].

To align the methodology with K10 studies in medical and clinical populations, the present study used K10 in three complementary ways. First, K10 was modeled continuously, per 5-point increase, to preserve information and avoid overreliance on arbitrary categories. Second, for descriptive tables, clinically familiar categories were used: <20 lower K10 category, 20–24 mild distress, 25–29 moderate distress, and ≥30 severe distress. Third, sensitivity analyses used Andrews and Slade categories of 10–15 low, 16–21 moderate, 22–29 high, and 30–50 very high distress [11]. A K10 score >20 was also recorded as a Turkish-validation-informed screening threshold because Altun et al. [12] reported strong diagnostic performance at this cutoff in the Turkish adaptation study. Throughout the manuscript, K10 was interpreted as a measure of non-specific psychological distress rather than as a formal diagnosis of anxiety or depression.

If one item was missing, the total score was prorated only when at least 9 of 10 items were completed and the missing item was not a skip-dependent item; otherwise, the questionnaire was repeated once during the same hospitalization. Patients with more than one missing item were excluded from the primary K10 analysis. In the final dataset, K10 completion was complete. Internal consistency of the scale in the cohort was assessed using Cronbach’s alpha; an alpha coefficient above 0.80 was considered acceptable for research use. K10 results were not used to determine the revascularization strategy during the index hospitalization.

### 2.5. Clinical, Laboratory, and Echocardiographic Variables

Demographic data, cardiovascular risk factors, medical history, medication use, vital signs, Killip class, laboratory parameters, echocardiographic findings, and angiographic characteristics were prospectively recorded using a standardized case report form. Hypertension was defined as prior diagnosis, use of antihypertensive medication, or repeated blood pressure values meeting guideline thresholds after stabilization. Diabetes mellitus was defined as prior diagnosis, use of glucose-lowering medication, or HbA1c meeting diagnostic criteria. Current smoking was defined as active tobacco use within the previous 30 days. Chronic kidney disease was defined as an estimated glomerular filtration rate <60 mL/min/1.73 m^2^ or a known history of chronic kidney disease. Prior psychiatric diagnosis, current antidepressant use, current anxiolytic use, sleep-related complaints when available, pain intensity at the time of K10 completion when available, opioid analgesic use, and the use of sedative medication during hospitalization were recorded to support sensitivity interpretation. These variables were not used to exclude patients unless reliable self-report was impossible. Creatinine clearance for the SYNTAX II calculation was estimated using the Cockcroft–Gault formula. Left ventricular ejection fraction was measured by transthoracic echocardiography during the index hospitalization using the biplane Simpson method when image quality allowed.

### 2.6. Coronary Angiography and Syntax Scoring

Coronary angiography was performed according to standard clinical practice through radial or femoral access. Significant coronary artery disease was defined as ≥50% diameter stenosis in a vessel with reference diameter ≥ 1.5 mm. This anatomical threshold of ≥50% was strictly utilized to comply with the official, standardized SYNTAX score algorithm, ensuring accurate and reproducible calculation. Multivessel disease was defined as significant stenosis in two or more major epicardial coronary arteries or their major branches. Unprotected left main disease was defined as ≥50% stenosis of the left main coronary artery without a patent bypass graft to the left anterior descending or circumflex territory.

The SYNTAX I score was calculated using the original SYNTAX algorithm by two experienced interventional cardiologists who were blinded to the K10 score, psychological data, and the statistical hypotheses of the study [4,5]. Lesion-level characteristics, including total occlusion, bifurcation, trifurcation, aorto-ostial disease, severe tortuosity, length >20 mm, heavy calcification, thrombus, and diffuse disease, were adjudicated from diagnostic angiographic projections before definitive intervention whenever possible. In STEMI, the initial diagnostic angiogram before balloon inflation or stenting was used for SYNTAX scoring. If the diagnostic view was inadequate because of acute occlusion, all available pre- and post-wiring views were reviewed to reconstruct the culprit anatomy without including procedural complications. In patients with previous CABG, graft lesions were not used to inflate the native-vessel SYNTAX score. These patients were retained only when native-vessel anatomy remained interpretable, and the robustness of the findings was tested by repeating the analyses after excluding prior CABG patients.

Disagreements between the two readers were handled using a prespecified adjudication protocol. If the absolute difference between readers was ≤5 points and both scores remained in the same risk category, the mean of the two values was used. If the difference was >5 points or resulted in discordant low/intermediate/high categories, a third senior interventional cardiologist adjudicated the final score. Interobserver reproducibility was assessed using the intraclass correlation coefficient for continuous SYNTAX I score and weighted kappa for SYNTAX category.

The SYNTAX II PCI score was calculated using the validated SYNTAX II variables: anatomical SYNTAX I score, age, sex, creatinine clearance, left ventricular ejection fraction, chronic obstructive pulmonary disease, peripheral vascular disease, and unprotected left main disease [7]. The PCI version of SYNTAX II was selected as the key clinical–anatomical score because all enrolled patients underwent invasive evaluation, and most ACS patients were considered for PCI during the index hospitalization. SYNTAX II CABG score was evaluated as an exploratory variable but was not the main endpoint of the study. Because SYNTAX II was originally developed to combine clinical and anatomical risk rather than to measure pure coronary anatomy, analyses involving SYNTAX II were interpreted as clinical–anatomical association analyses.

### 2.7. Study Endpoints

The primary endpoint was intermediate/high anatomical complexity, defined as a SYNTAX I score ≥ 23. This threshold was chosen because it separates low anatomical complexity from intermediate and high categories in the conventional SYNTAX framework. Continuous SYNTAX I score was also retained as a major endpoint to avoid loss of information caused by dichotomization. The key secondary endpoint was continuous SYNTAX II PCI score. High clinical–anatomical complexity, defined as a SYNTAX II PCI score ≥ 36, was used as an additional binary endpoint. Because there is no universally accepted ACS-specific SYNTAX II PCI cutoff, this binary threshold was treated as a cohort-based high-risk definition rather than as a universal diagnostic threshold. Specifically, this cutoff (≥36) was selected based on the upper tertile distribution of our specific cohort to identify a high-risk stratum. Therefore, its clinical implications should be interpreted cautiously. Additional secondary endpoints were SYNTAX II CABG score, multivessel disease, and unprotected left main disease.

### 2.8. Statistical Analysis

Statistical analyses were performed using IBM SPSS Statistics for Windows, version 28.0 (IBM Corp., Armonk, NY, USA), and R software, version 4.3.2 (R Foundation for Statistical Computing, Vienna, Austria). SPSS was used for data cleaning, descriptive statistics, group comparisons, correlation analyses, and conventional regression models. R was used for receiver operating characteristic (ROC) analysis, DeLong comparisons, restricted cubic spline modeling, calibration analysis, bootstrap optimism correction, cross-validation, net reclassification improvement, integrated discrimination improvement, and graphical diagnostics, using appropriate packages including pROC, rms, boot, caret, and PredictABEL where applicable. Continuous variables were reported as mean ± standard deviation or median with interquartile range according to distribution, and categorical variables were reported as number and percentage. Normality was assessed using histograms, Q-Q plots, and the Shapiro–Wilk test. Comparisons across K10 categories were performed using one-way analysis of variance or the Kruskal–Wallis test for continuous variables and the chi-squared test or Fisher exact test for categorical variables. A linear trend across ordered K10 categories was examined when appropriate.

Correlations between K10 and continuous or ordinal variables, including SYNTAX I and SYNTAX II PCI scores, were assessed using Spearman rank correlation because these measures are often non-normally distributed and may contain outliers. Associations between K10 and binary angiographic variables, such as multivessel disease, chronic total occlusion, and severe calcification, were evaluated using point-biserial correlation. Multivariable linear regression was used to examine the association between K10 and continuous SYNTAX I and SYNTAX II PCI scores, and multivariable logistic regression was used for SYNTAX I ≥ 23 and SYNTAX II PCI ≥ 36. K10 was entered as the main exposure per 5-point increase to preserve clinical interpretability. Covariates were selected a priori according to clinical relevance and previous ACS/SYNTAX literature rather than by univariable *p*-value screening. A hierarchical adjustment strategy was used. Model 1 was defined as a basic cardiovascular risk model and included age, sex, body mass index, diabetes mellitus, hypertension, current smoking, family history of premature coronary artery disease, and LDL-C level. Model 2 was defined as an extended clinical model and included all Model 1 variables plus previous myocardial infarction, previous PCI, previous CABG, chronic kidney disease, left ventricular ejection fraction, and ACS subtype. Model 3 was reserved for prespecified psychocardiologic and timing sensitivity analyses and added prior psychiatric diagnosis, antidepressant/anxiolytic use, K10 timing relative to angiography, pain intensity at the time of K10 completion when available, and opioid/sedative exposure when available. Model 1 and Model 2 constituted the main adjustment framework, whereas Model 3 was used only for prespecified sensitivity analyses and is therefore reported separately in the sensitivity analysis table.

For SYNTAX I analyses, variables that are direct components or downstream expressions of the anatomical SYNTAX score, such as multivessel disease, bifurcation lesion, severe calcification, chronic total occlusion, and lesion length, were not entered into the main adjusted models to avoid overadjustment. For SYNTAX II PCI analyses, the endpoint was interpreted as a clinical–anatomical construct rather than a pure anatomical outcome. Because several clinical variables are mathematical components of the SYNTAX II score, component-overlapping models were not interpreted as independent etiologic prediction models. Instead, they were considered exploratory, component-enriched discrimination analyses designed to test whether K10 contributed information beyond the clinical–anatomical framework.

Odds ratios were reported with 95% confidence intervals. Linearity of K10 in the logit was evaluated using restricted cubic splines and by comparing models with K10 categories. Multicollinearity was assessed with the variance inflation factor, and VIF <2.5 was considered acceptable. Model fit and calibration were evaluated using calibration intercept, calibration slope, Brier score, and visual calibration plots when appropriate. Discrimination was evaluated using ROC curves and AUC. The K10-alone ROC curves were constructed directly from the observed integer K10 score, whereas model-based ROC curves were constructed from predicted probabilities. Optimal K10 thresholds were identified using the Youden index, but these thresholds were considered exploratory and not diagnostic cutoffs.

Incremental predictive information was tested by comparing the extended clinical model with and without K10. AUCs were compared using DeLong testing. Internal validation was performed using five-fold cross-validation and bootstrap optimism correction with 1000 resamples. Cross-validated ROC curves were constructed from pooled out-of-fold predicted probabilities, so each patient contributed a prediction generated from a model in which that patient was not used for training. This distinction was important because the K10-alone ROC analysis was based on a discrete integer score, whereas the model-based ROC curves were based on continuous predicted probabilities and therefore may show a different stepwise appearance. Bootstrap-corrected AUC was calculated by subtracting estimated optimism from the apparent AUC. Net reclassification improvement and integrated discrimination improvement were calculated as complementary exploratory indices rather than as definitive evidence for a deployable risk calculator.

Prespecified sensitivity analyses included K10 as categorical strata, the Turkish-validation-informed threshold (>20), Andrews-Slade categories, ACS subtype interaction testing, exclusion of patients with previous CABG, additional adjustment for prior psychiatric diagnosis and antidepressant/anxiolytic use, restriction to patients whose K10 was completed before angiography when applicable, and interpretation of SYNTAX II models with and without mathematical component overadjustment. Missing covariate data were assessed before modeling; because missingness was low, complete-case analysis was used for the primary models. All tests were two-sided, and *p* < 0.05 was considered statistically significant.

## 3. Results

Between 6 January 2026 and 5 June 2026, 865 patients with suspected or confirmed ACS were assessed for eligibility. Of these, 103 patients were excluded before analytic eligibility because they did not undergo invasive coronary angiography, had an alternative final diagnosis or non-atherothrombotic myocardial injury, refused or were unable to provide informed consent, had persistent instability, cardiogenic shock, mechanical ventilation, neurocognitive or communication barriers, active psychosis or manic episode, sedative/analgesic exposure precluding reliable self-report, or outside-hospital culprit intervention without diagnostic angiographic images. Among 762 patients who underwent K10 assessment and angiographic review, 12 were further excluded because of incomplete or unreliable K10 assessment, inadequate angiographic projections for SYNTAX scoring, or missing variables required for SYNTAX II PCI calculation. The final analytic cohort included 750 consecutive eligible ACS patients with complete K10, SYNTAX I, and SYNTAX II PCI data (Figure 1).

In the final analytic cohort, STEMI was present in 337 patients (44.9%), NSTEMI in 310 (41.3%), and USAP in 103 (13.7%). The mean age was 61.3 ± 11.6 years, and 543 patients (72.4%) were men (Table 1). Diabetes mellitus was present in 205 patients (27.3%), hypertension in 393 (52.4%), current smoking in 286 (38.1%), chronic kidney disease in 53 (7.1%), and the mean left ventricular ejection fraction was 49.5 ± 7.3% (Table 1 and Table 2).

The mean K10 score was 23.7 ± 6.7. Using the clinical descriptive categories, 197 patients (26.3%) had lower K10 category (<20), 217 (28.9%) mild distress (20–24), 203 (27.1%) moderate distress (25–29), and 133 (17.7%) severe distress (≥30). Internal consistency of the K10 in this study cohort was high (Cronbach’s alpha 0.91). Prior depression or anxiety diagnosis, antidepressant use, and anxiolytic use were more frequent across increasing K10 categories, as expected, but the association between K10 and SYNTAX-based endpoints persisted after additional adjustment for these variables. Patients with higher K10 scores tended to have more diabetes, hypertension, higher hs-CRP, longer symptom-to-door time, and higher prevalence of multivessel disease, although age and sex distribution were similar across categories (Table 1, Table 2, Table 3 and Table 4).

The mean SYNTAX I score was 22.4 ± 9.5. Low, intermediate, and high anatomical complexity were observed in 363 (48.4%), 266 (35.5%), and 121 (16.1%) patients, respectively. The mean SYNTAX II PCI score was 31.2 ± 9.6, and 220 patients (29.3%) had a SYNTAX II PCI score ≥36. Across increasing K10 categories, the mean SYNTAX I score increased from 17.2 ± 8.7 to 28.4 ± 8.5, and the mean SYNTAX II PCI score increased from 27.0 ± 8.2 to 36.7 ± 9.4 (both *p* < 0.001) (Table 4). Interobserver reproducibility for SYNTAX I scoring was consistently excellent across all ACS subgroups (STEMI, NSTEMI, and USAP) (intraclass correlation coefficient 0.92), and agreement for SYNTAX category was good (weighted kappa 0.84).

The K10 score showed a moderate positive correlation with the SYNTAX I score (Spearman rho = 0.417, *p* < 0.001) and a weaker but significant positive correlation with the SYNTAX II PCI score (rho = 0.348, *p* < 0.001) (Table 5). In multivariable linear regression, each 5-point increase in K10 was associated with a 2.57-point higher SYNTAX I score (95% CI 2.15–3.00; *p* < 0.001) and a 2.11-point higher SYNTAX II PCI score (95% CI 1.84–2.39; *p* < 0.001) (Table 6).

**Table 5:** Correlation of K10 score with anatomical, clinical, laboratory, and risk-score variables.

Variable Correlated with K10	Correlation Method	Crude Coefficient	*p* Value	Age/Sex-Adjusted Coefficient	*p* Value	Fully Adjusted Coefficient *	*p* Value
SYNTAX I score	Spearman rho	0.417	<0.001	0.405	<0.001	0.332	<0.001
SYNTAX II PCI score	Spearman rho	0.348	<0.001	0.336	<0.001	0.274	<0.001
SYNTAX II CABG score	Spearman rho	0.326	<0.001	0.319	<0.001	0.248	<0.001
GRACE risk score	Spearman rho	0.281	<0.001	0.238	<0.001	0.171	<0.001
Number of diseased vessels	Spearman rho	0.311	<0.001	0.300	<0.001	0.244	<0.001
Multivessel disease	Point-biserial r	0.262	<0.001	0.251	<0.001	0.202	<0.001
Chronic total occlusion	Point-biserial r	0.151	<0.001	0.145	<0.001	0.111	0.006
Severe calcification	Point-biserial r	0.174	<0.001	0.160	<0.001	0.119	0.003
hs-CRP	Spearman rho	0.236	<0.001	0.229	<0.001	0.166	<0.001
Peak troponin I	Spearman rho	0.116	0.002	0.107	0.004	0.068	0.070
LVEF	Spearman rho	−0.031	0.397	−0.025	0.498	−0.019	0.602
Creatinine clearance	Spearman rho	−0.092	0.012	−0.071	0.052	−0.038	0.299
Symptom-to-door time	Spearman rho	0.188	<0.001	0.181	<0.001	0.133	0.001
Length of stay	Spearman rho	0.201	<0.001	0.188	<0.001	0.125	0.002

* Fully adjusted partial correlations were adjusted according to the extended clinical covariate set: age, sex, body mass index, diabetes mellitus, hypertension, LDL-C level, current smoking, family history of premature CAD, previous myocardial infarction, previous PCI/CABG, chronic kidney disease, LVEF, and ACS subtype. ACS, acute coronary syndrome; CABG, coronary artery bypass grafting; hs-CRP, high-sensitivity C-reactive protein; LDL-C, low-density lipoprotein cholesterol; LVEF, left ventricular ejection fraction; PCI, percutaneous coronary intervention.

**Table 6:** Regression analyses evaluating K10 as a predictor of continuous and categorical coronary complexity.

Endpoint/Model	K10 Expression	Unadjusted Estimate (95% CI)	*p* Value	Model 1 Adjusted Estimate (95% CI)	*p* Value	Model 2 Adjusted Estimate (95% CI)	*p* Value
Linear: SYNTAX I score	Per 5-point increase	3.26 (2.86–3.66)	<0.001	2.86 (2.45–3.27)	<0.001	2.57 (2.15–3.00)	<0.001
Linear: SYNTAX I score	Mild vs. low/no	4.61 (3.12–6.10)	<0.001	4.02 (2.58–5.46)	<0.001	3.48 (2.02–4.94)	<0.001
Linear: SYNTAX I score	Moderate vs. low/no	7.11 (5.58–8.64)	<0.001	6.21 (4.74–7.68)	<0.001	5.36 (3.86–6.86)	<0.001
Linear: SYNTAX I score	Severe vs. low/no	11.20 (9.48–12.92)	<0.001	9.78 (8.12–11.44)	<0.001	8.21 (6.49–9.93)	<0.001
Linear: SYNTAX II PCI score	Per 5-point increase	2.68 (2.30–3.06)	<0.001	2.36 (2.01–2.71)	<0.001	2.11 (1.84–2.39)	<0.001
Linear: SYNTAX II PCI score	Severe vs. low/no	9.70 (7.82–11.58)	<0.001	8.14 (6.40–9.88)	<0.001	6.72 (5.04–8.40)	<0.001
Logistic: SYNTAX I ≥23	Per 5-point increase	2.17 (1.91–2.46)	<0.001	1.97 (1.70–2.28)	<0.001	1.84 (1.59–2.12)	<0.001
Logistic: SYNTAX I ≥23	K10 ≥ 24	3.42 (2.46–4.75)	<0.001	2.91 (2.04–4.15)	<0.001	2.57 (1.78–3.72)	<0.001
Logistic: SYNTAX I ≥23	Severe vs. low/no	8.88 (5.47–14.41)	<0.001	7.02 (4.19–11.77)	<0.001	5.74 (3.33–9.89)	<0.001
Logistic: SYNTAX II PCI ≥36	Per 5-point increase	2.45 (2.06–2.91)	<0.001	2.31 (1.91–2.79)	<0.001	2.26 (1.85–2.75)	<0.001
Logistic: SYNTAX II PCI ≥36	K10 ≥ 26	3.30 (2.34–4.66)	<0.001	3.06 (2.10–4.46)	<0.001	2.91 (1.95–4.35)	<0.001
Logistic: SYNTAX II PCI ≥36	Severe vs. low/no	5.77 (3.54–9.41)	<0.001	5.39 (3.17–9.17)	<0.001	4.87 (2.78–8.53)	<0.001

Linear models are presented as beta coefficients; logistic models are presented as odds ratios. Model 1 was the basic cardiovascular risk model and adjusted for age, sex, body mass index, diabetes mellitus, hypertension, LDL-C level, current smoking, and family history of premature CAD. Model 2 was the extended clinical model and adjusted for Model 1 covariates plus previous myocardial infarction, previous PCI, previous CABG, chronic kidney disease, LVEF, and ACS subtype. Model 3 was not included in this main regression table because it was prespecified as a psychocardiologic/timing sensitivity model; corresponding Model 3 estimates are presented in Table 7. For SYNTAX II endpoints, component-overlapping adjustment was interpreted cautiously because several clinical variables are mathematical components of SYNTAX II. CI, confidence interval; K10, Kessler Psychological Distress Scale-10; LVEF, left ventricular ejection fraction; PCI, percutaneous coronary intervention.

In multivariable logistic regression, K10 remained independently associated with both higher-complexity endpoints (Table 6). Each 5-point increase in K10 was associated with SYNTAX I ≥23 (extended model-adjusted OR 1.84, 95% CI 1.59–2.12; *p* < 0.001) and SYNTAX II PCI ≥36 (extended model-adjusted OR 2.26, 95% CI 1.85–2.75; *p* < 0.001). The association was directionally consistent in STEMI, NSTEMI, and USAP subgroups, without significant interaction by ACS subtype. Results were materially unchanged when prior CABG patients were excluded, when prior psychiatric diagnosis and antidepressant/anxiolytic use were added to the models, and when analyses were restricted to patients whose K10 was completed before angiography (Table 7).

**Table 7:** Prespecified sensitivity analyses evaluating robustness of the K10-SYNTAX association.

Sensitivity Analysis	Endpoint	K10 Expression	Adjusted Estimate (95% CI)	*p* Value
Excluding previous CABG patients (n = 714)	SYNTAX I ≥ 23	Per 5-point increase	OR 1.79 (1.54–2.08)	<0.001
Excluding previous CABG patients (n = 714)	SYNTAX II PCI ≥ 36	Per 5-point increase	OR 2.18 (1.77–2.68)	<0.001
Additional adjustment for psychiatric history and antidepressant/anxiolytic use	SYNTAX I ≥ 23	Per 5-point increase	OR 1.78 (1.53–2.07)	<0.001
Additional adjustment for psychiatric history and antidepressant/anxiolytic use	SYNTAX II PCI ≥ 36	Per 5-point increase	OR 2.12 (1.72–2.61)	<0.001
K10 completed before angiography subgroup (n = 413)	SYNTAX I ≥ 23	Per 5-point increase	OR 1.72 (1.41–2.11)	<0.001
K10 completed before angiography subgroup (n = 413)	SYNTAX II PCI ≥ 36	Per 5-point increase	OR 1.98 (1.50–2.61)	<0.001
Andrews-Slade K10 categories	SYNTAX I ≥ 23	Very high vs. low/moderate	OR 4.92 (3.04–7.96)	<0.001
Andrews-Slade K10 categories	SYNTAX II PCI ≥ 36	Very high vs. low/moderate	OR 4.31 (2.54–7.31)	<0.001
Turkish-validation-informed threshold	SYNTAX I ≥ 23	K10 > 20	OR 2.39 (1.62–3.53)	<0.001
Turkish-validation-informed threshold	SYNTAX II PCI ≥ 36	K10 > 20	OR 2.05 (1.31–3.20)	0.001
ACS subtype interaction	SYNTAX I ≥ 23	K10 x ACS subtype	*p* for interaction = 0.418	0.418
Model 3 psychocardiologic sensitivity model	SYNTAX I ≥ 23	Per 5-point increase	OR 1.74 (1.48–2.05)	<0.001
Model 3 psychocardiologic sensitivity model	SYNTAX II PCI ≥ 36	Per 5-point increase	OR 2.04 (1.63–2.55)	<0.001
Restricted cubic spline	SYNTAX I ≥ 23	K10 continuous	*p* for non-linearity = 0.928	0.928
Restricted cubic spline	SYNTAX II PCI ≥ 36	K10 continuous	*p* for non-linearity = 0.881	0.881

CABG, coronary artery bypass grafting; K10, Kessler Psychological Distress Scale-10; OR, odds ratio; PCI, percutaneous coronary intervention. Unless otherwise specified, estimates are adjusted according to the extended clinical model. Psychiatric/timing sensitivity models additionally considered prior psychiatric diagnosis, antidepressant/anxiolytic use, K10 timing relative to angiography, pain intensity when available, and opioid/sedative exposure when available.

In sensitivity analyses, K10 remained associated with SYNTAX I ≥23 and SYNTAX II PCI ≥36 after excluding prior CABG patients (OR 1.79, 95% CI 1.54–2.08 and OR 2.18, 95% CI 1.77–2.68), after additional adjustment for prior psychiatric diagnosis and antidepressant/anxiolytic use (OR 1.78, 95% CI 1.53–2.07 and OR 2.12, 95% CI 1.72–2.61), and in the subgroup whose K10 was completed before angiography (n = 413; OR 1.72, 95% CI 1.41–2.11 and OR 1.98, 95% CI 1.50–2.61). Alternative K10 classifications produced similar findings for both endpoints, including Andrews–Slade categories and the Turkish-validation-informed K10 > 20 threshold (Table 7). Restricted cubic spline analyses did not show significant departure from linearity for the association of K10 with SYNTAX I ≥23 (*p* for non-linearity = 0.928) or SYNTAX II PCI ≥36 (*p* for non-linearity = 0.881).

**Figure 2:**
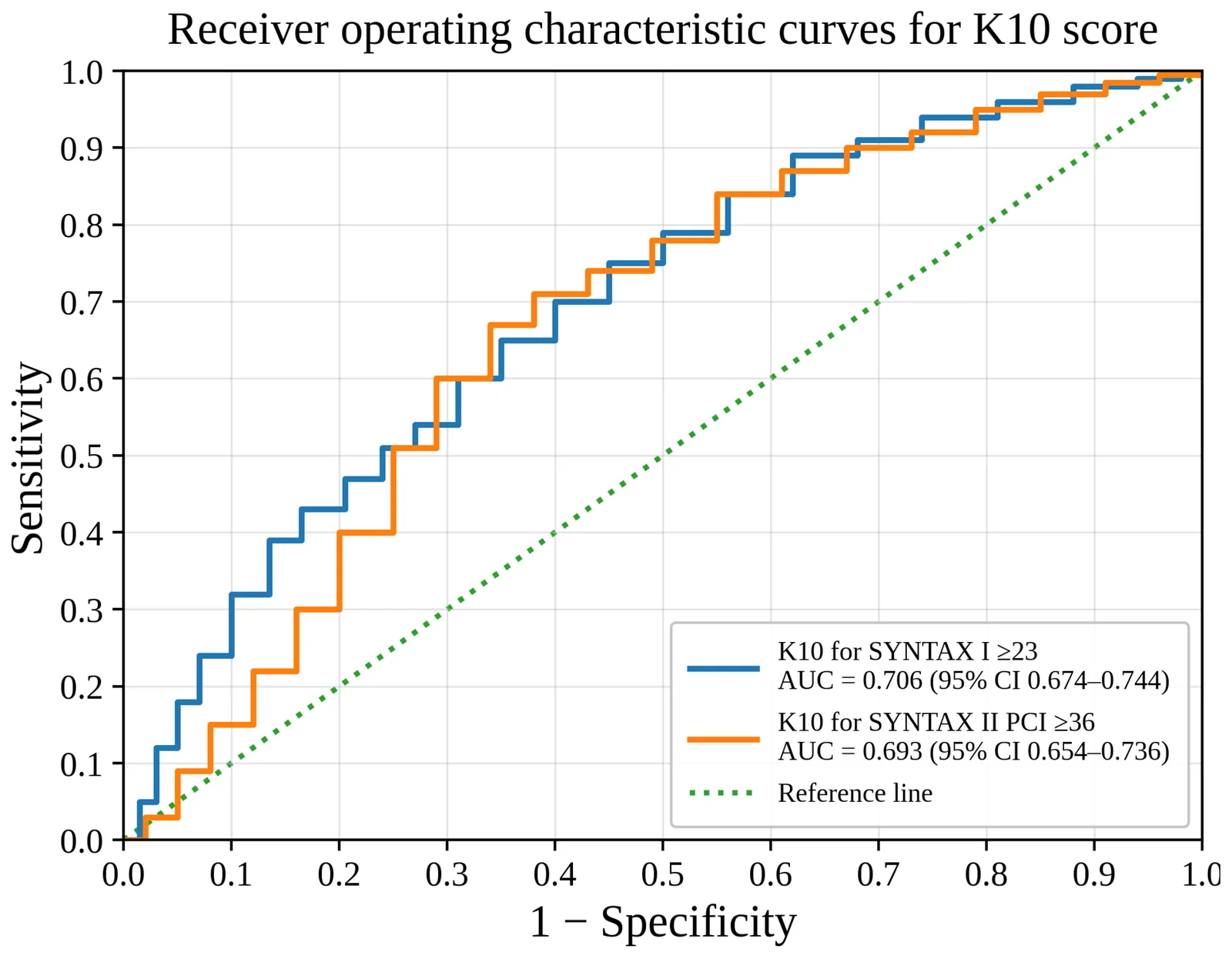
Receiver operating characteristic curves showing the ability of the K10 score alone to discriminate intermediate/high anatomical complexity (SYNTAX I ≥ 23) and high clinical–anatomical complexity (SYNTAX II PCI ≥ 36). Because K10 is a discrete integer score, ROC curves are displayed in a stepwise format. AUC values and 95% confidence intervals are presented in the legend.

**Figure 3:**
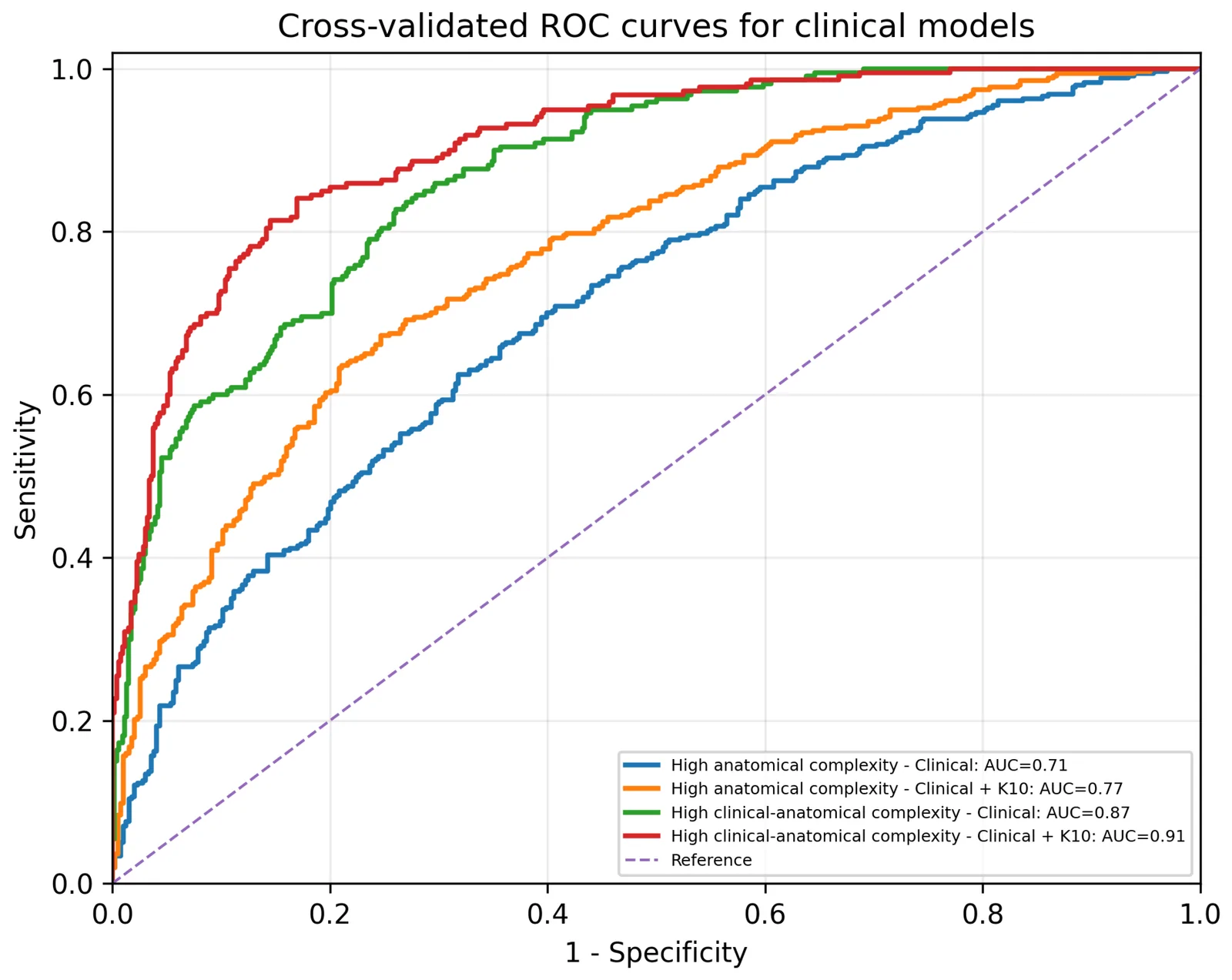
Cross-validated receiver operating characteristic curves for extended clinical models with and without K10, constructed from pooled out-of-fold predicted probabilities. Addition of K10 improved cross-validated discrimination for both SYNTAX I ≥ 23 and SYNTAX II PCI ≥ 36. For SYNTAX II PCI ≥ 36, model ROC curves should be interpreted as component-enriched exploratory discrimination analyses because SYNTAX II incorporates clinical variables that overlap with the prediction model. These analyses were not intended to define a definitive clinical risk-score model.

In ROC analysis, K10 alone discriminated SYNTAX I ≥23 with an AUC of 0.706 (95% CI 0.674–0.744) (Figure 2, Table 8). The optimal K10 cutoff was ≥24, with sensitivity 65.3% and specificity 64.6%. For SYNTAX II PCI ≥36, K10 had an AUC of 0.693 (95% CI 0.654–0.736), and the optimal cutoff was ≥26 with sensitivity 60.0% and specificity 70.0%. These K10-alone ROC curves were displayed in a stepwise format because K10 is a discrete integer score rather than a continuous predicted probability. Adding K10 to the extended clinical model improved cross-validated AUC for SYNTAX I ≥23 from 0.708 to 0.774 and for SYNTAX II PCI ≥36 from 0.871 to 0.905 (Figure 3, Table 8). The corresponding bootstrap optimism-corrected AUCs for the extended clinical model plus K10 were 0.754 and 0.899, respectively. Cross-validated model ROC curves were based on pooled out-of-fold predicted probabilities. Calibration remained acceptable after the addition of K10, with calibration slopes close to 1.0 and lower Brier scores than the extended clinical models alone. DeLong comparisons, NRI, and IDI supported incremental predictive information from K10 (Table 8). For SYNTAX II PCI ≥36, however, the model-based ROC curves were interpreted as component-enriched exploratory discrimination analyses because SYNTAX II incorporates several clinical variables that overlap with the clinical model. Therefore, these analyses were not interpreted as the development of an independent clinical risk score.

**Table 8:** ROC analysis, calibration, and internal validation of the K10 and clinical prediction models.

Endpoint/Model	AUC (95% CI)	Cutoff	Sensitivity	Specificity	PPV	NPV	Youden Index	Brier Score	Calibration Slope	CV-AUC	Optimism-Corrected AUC
K10 alone: SYNTAX I ≥23	0.706 (0.674–0.744)	≥24	65.3%	64.6%	66.3%	63.4%	0.299	0.223	-	0.699	0.697
K10 alone: SYNTAX II PCI ≥36	0.693 (0.654–0.736)	≥26	60.0%	70.0%	45.4%	80.8%	0.300	0.185	-	0.686	0.681
Clinical model: SYNTAX I ≥23	0.708 (0.671–0.745)	-	-	-	-	-	-	0.216	0.940	0.708	0.688
Clinical model + K10: SYNTAX I ≥23	0.774 (0.740–0.808)	-	-	-	-	-	-	0.193	0.950	0.774	0.754
Delta AUC/NRI/IDI: SYNTAX I ≥23	+0.066	-	-	-	-	-	-	-	-	NRI 0.261	IDI 0.071
Clinical model: SYNTAX II PCI ≥36	0.871 (0.842–0.900)	-	-	-	-	-	-	0.129	0.963	0.871	0.862
Clinical model + K10: SYNTAX II PCI ≥36	0.905 (0.881–0.929)	-	-	-	-	-	-	0.108	0.961	0.905	0.899
Delta AUC/NRI/IDI: SYNTAX II PCI ≥36	+0.034	-	-	-	-	-	-	-	-	NRI 0.214	IDI 0.052

The extended clinical model used for incremental prediction was the extended clinical model: age, sex, body mass index, diabetes mellitus, hypertension, LDL-C level, current smoking, family history of premature CAD, previous myocardial infarction, previous PCI/CABG, chronic kidney disease, LVEF, and ACS subtype. Cross-validated ROC curves were drawn from pooled out-of-fold predicted probabilities. For SYNTAX II PCI ≥ 36, clinical prediction curves were interpreted as component-enriched exploratory discrimination analyses because SYNTAX II incorporates clinical variables that overlap with the model; these analyses were not considered an independent clinical risk-score model. AUC, area under the curve; CI, confidence interval; IDI, integrated discrimination improvement; NRI, net reclassification improvement; ROC, receiver operating characteristic.

## 4. Discussion

In this prospective ACS cohort, K10-defined psychological distress was independently associated with higher SYNTAX I and SYNTAX II PCI scores. This relationship was observed in continuous analyses, categorical K10 strata, multivariable regression, ROC analysis, and internally validated incremental prediction models. Importantly, the association persisted in sensitivity analyses excluding previous CABG patients, adjusting for psychiatric history and related medication use, and evaluating the subgroup in whom K10 was completed before angiography. These findings support the rationale for integrating brief psychological distress screening into ACS research and potentially into post-ACS care pathways, while emphasizing that external validation is required before K10 can be used as a formal risk-stratification component.

This distinction is important for the present study. We did not treat K10 as a formal psychiatric diagnosis, nor did we assume that the scale captures only acute anxiety. Instead, we used it as a standardized measure of recent distress burden, including anxiety- and depression-related symptoms over the preceding four weeks. This is methodologically more appropriate than describing K10 as a purely acute anxiety score.

The Turkish adaptation provides further justification for use in this cohort. Altun et al. [12] translated the K10 according to World Health Organization World Mental Health Initiative translation principles and reported excellent internal consistency, high test–retest reliability, and strong ROC performance in Turkish clinical settings. Therefore, the validated Turkish version of the K10 scale is a suitable instrument for cardiology research in Turkiye. At the same time, the present protocol deliberately added ACS-specific safeguards: the scale was administered only after hemodynamic stabilization, not before reperfusion in STEMI, and not during uncontrolled pain or delirium. These details are essential because acute ischemic pain, fear, opioids, sedatives, and critical illness could distort patient-reported psychological responses. Recording whether K10 was administered before or after angiography and testing a pre-angiography subgroup strengthened interpretability because knowledge of angiographic severity may itself increase distress.

The association between psychological distress and coronary complexity has prior but limited support. Kaya et al. [22] studied ACS patients undergoing PCI and reported that anxiety and depression levels measured by HADS were associated with SYNTAX score. Their work is particularly relevant because it connected a psychological scale with objective angiographic burden in an ACS population. The present study extends that idea in several important ways: it uses a larger sample size, includes the entire ACS spectrum, uses K10 rather than HADS, includes blinded dual-reader SYNTAX scoring, evaluates both anatomical SYNTAX I and clinical–anatomical SYNTAX II PCI, and provides calibration and internal validation analyses. Ekici et al. [23] similarly linked emotional status and health-related quality of life with coronary artery disease severity, supporting the broader concept that psychological variables are not independent of cardiovascular disease expression. Thus, the novelty of the present work is not simply the use of another psychological questionnaire but the integration of a validated distress scale with both anatomical and clinical–anatomical coronary complexity.

The SYNTAX I result is clinically intuitive. Diffuse atherosclerosis, multivessel disease, calcification, bifurcation lesions, and chronic total occlusions are markers of long-standing coronary pathology. Psychological distress could contribute to this pattern through chronic neuroendocrine and behavioral pathways. Repeated sympathetic activation may increase heart rate, blood pressure variability, vascular tone, and myocardial oxygen demand. HPA-axis activation may promote metabolic dysregulation, insulin resistance, visceral adiposity, and inflammatory signaling. Distress-related sleep disruption may worsen autonomic balance, blood pressure control, and glycemic variability. These pathways are not specific to ACS but may contribute over time to atherosclerotic progression and plaque vulnerability.

Inflammation represents another plausible biological bridge. Patients with higher K10 scores in the present cohort had higher hs-CRP values, and this is consistent with the idea that psychological distress may be associated with low-grade inflammatory activation. Inflammation participates in endothelial dysfunction, plaque progression, plaque rupture, thrombus formation, and adverse remodeling after myocardial infarction. Distress may also influence platelet activity, catecholamine-mediated thrombogenicity, and microvascular dysfunction. Although the present study was not designed to prove mediation, these pathways provide a coherent framework for why a distress scale could correlate with angiographic complexity.

Behavioral mechanisms should not be underestimated. Patients with higher psychological distress may smoke more, exercise less, sleep poorly, delay seeking care, show lower adherence to antihypertensive, lipid-lowering, and antidiabetic medication, and participate less in preventive health services. They may also have lower socioeconomic resources or reduced social support, both of which can influence risk-factor control and treatment access. These variables are difficult to fully capture in a single-center cardiology dataset. Therefore, K10 may function as a summary marker of biological, behavioral, and social vulnerability rather than as a direct causal factor by itself.

The SYNTAX II finding is more complex. Therefore, an association between K10 and SYNTAX II may reflect both coronary anatomy and systemic clinical vulnerability. In the present models, K10 remained associated with SYNTAX II PCI even after adjustment. However, interpretation must be cautious because several conventional clinical covariates are also mathematical components of SYNTAX II itself. For that reason, the statistical plan prioritized hierarchical clinical association models and treated component-enriched SYNTAX II prediction models as exploratory sensitivity analyses rather than as independent diagnostic risk tools. This approach is methodologically important because it reduces the risk of artificially strengthening or weakening the K10-SYNTAX II relationship through mathematical coupling.

The ROC findings deserve balanced interpretation. K10 alone had moderate discrimination for SYNTAX I ≥ 23 and SYNTAX II PCI ≥ 36. Because K10 is a discrete integer score, its ROC curves appropriately have a stepwise appearance and should be interpreted as threshold-based operating characteristics rather than as a smooth continuous-risk function. AUC values around 0.69–0.71 do not support using K10 as a stand-alone anatomical risk tool. A patient should never undergo or avoid angiography based on K10. However, the improvement in AUC when K10 was added to clinical variables suggests incremental information. This is clinically plausible: K10 captures a dimension that is not fully represented by age, diabetes, smoking, LDL-C, renal function, ejection fraction, or ACS subtype. Therefore, K10 may be useful as a low-cost adjunctive marker, especially for identifying patients who need integrated psychological and preventive support.

Internal validation strengthens the model but does not replace external validation. Five-fold cross-validation was based on pooled out-of-fold predicted probabilities, and bootstrap optimism correction showed stable AUC estimates. Accordingly, the model ROC curves are expected to appear more granular than the K10-alone ROC curves because they are based on continuous predicted probabilities rather than a single discrete questionnaire score. Calibration slopes remained close to 1.0 after adding K10 to clinical variables, and the lower Brier scores in the combined models suggested improved overall prediction error. For SYNTAX II PCI ≥ 36, these ROC curves should be viewed as component-enriched exploratory discrimination analyses because the endpoint itself contains clinical variables that overlap with the prediction model. Thus, the analyses support incremental information from K10 but should not be interpreted as a ready-to-use clinical risk calculator. External validation in an independent ACS cohort would be required before any K10-based risk algorithm could be proposed. Multicenter validation would be particularly important because distress levels, symptom reporting, ACS presentation delay, and revascularization patterns may vary by region, culture, socioeconomic status, and healthcare access.

The practical implication is not that cardiologists should become psychiatrists, but that ACS pathways may benefit from structured psychological screening. K10 can be completed in a few minutes and scored easily. In STEMI, it should be administered after reperfusion and stabilization so it does not delay primary PCI. In NSTEMI and USAP, it can be administered after initial stabilization, ideally before detailed angiographic risk discussions. Patients with severe distress could be referred for psychiatric or psychological evaluation, sleep assessment, smoking cessation support, cardiac rehabilitation engagement, and closer follow-up. The most important clinical use may be secondary prevention rather than prediction of coronary anatomy.

The bidirectional nature of the association must be emphasized. It is possible that patients with more complex coronary disease experience more severe symptoms, longer hospitalization, more procedures, and greater fear, which then raises K10 scores. It is also possible that chronic distress contributes to atherosclerotic burden. A single early K10 measurement cannot fully distinguish these directions. Serial K10 measurements before discharge, at 1 month, and at 6 months would help separate persistent psychological vulnerability from transient event-related distress. Future studies should also include structured psychiatric interviews, prior psychiatric history, sleep quality, pain intensity, socioeconomic variables, and inflammatory biomarkers to clarify mechanisms.

Taken together, the present study provides a methodologically strong framework for investigating psychocardiological risk in ACS. Its central message is not that distress causes complex coronary anatomy in a direct linear manner, but that psychological distress is sufficiently connected with anatomical and clinical–anatomical risk to deserve systematic measurement. K10 may be a practical bridge between coronary risk stratification and comprehensive post-ACS care.

It is crucial to emphasize that the relationship between psychological distress and coronary complexity is bidirectional. While chronic psychological stress may drive accelerated atherosclerosis, severe coronary disease can amplify acute psychological distress due to symptom burden. Furthermore, identifying this psychocardiological phenotype using the K10 provides incremental value beyond traditional risk scores like GRACE or SYNTAX by facilitating targeted, multidisciplinary psychological interventions early in the post-ACS period. Future longitudinal studies are needed to evaluate the prognostic value of K10 in predicting long-term major adverse cardiovascular events (MACE) and to clarify the impact of post-discharge psychological trajectories.

### Limitations

Several limitations should be considered. First, this is a single-center design. Therefore, the findings may not generalize to centers with different ACS referral patterns, primary PCI volumes, socioeconomic catchment areas, or psychiatric service availability. Furthermore, cultural differences in symptom reporting might affect external validity, necessitating validation across diverse healthcare systems and ethnic populations. Second, K10 is a screening measure of non-specific psychological distress and cannot diagnose major depressive disorder, generalized anxiety disorder, panic disorder, acute stress disorder, or post-traumatic stress disorder. The scale should therefore be interpreted as a distress marker rather than as a diagnostic psychiatric endpoint. Third, because several K10 items may overlap with somatic symptoms experienced by patients with ACS, particularly fatigue, restlessness, and effortfulness, some degree of symptom contamination cannot be excluded despite administration after stabilization. Although pain control was required before questionnaire completion, pain intensity and opioid analgesic exposure may still have influenced self-reported distress in some patients.

Fourth, K10 evaluates symptoms during the previous four weeks, whereas ACS hospitalization may alter recall, emotional salience, and response style. Administering the questionnaire after stabilization and performing timing-based sensitivity analyses reduce acute pain-related and angiography-related bias but cannot eliminate the psychological impact of the event. Fifth, the temporal relationship remains uncertain. Higher K10 may precede ACS and contribute to disease progression, or more severe coronary disease may increase distress through symptom severity and perceived threat. Serial pre-event data are usually unavailable in ACS cohorts, and future studies should consider repeated K10 assessments after discharge.

Sixth, residual confounding is likely. Socioeconomic status, education, employment stress, social support, sleep apnea, insomnia, chronic pain, frailty, prior psychiatric diagnosis, antidepressant or anxiolytic use, medication adherence, diet, physical activity, and time from symptom onset to hospital admission may influence both distress and coronary complexity. Seventh, SYNTAX II contains clinical variables that overlap with conventional cardiovascular risk adjustment. Statistical models for SYNTAX II must therefore avoid inappropriate overadjustment and mathematical coupling. Eighth, SYNTAX I scoring after prior CABG is less standardized; although native-vessel scoring was used and sensitivity analyses excluding prior CABG patients showed consistent results, this subgroup should still be interpreted cautiously. Ninth, the present study focused on angiographic and clinical–anatomical complexity rather than longitudinal outcomes. Therefore, the relationship between K10 and post-discharge MACE, recurrent myocardial infarction, repeat revascularization, heart failure hospitalization, or mortality requires future follow-up studies. Finally, internal validation reduces overfitting concerns but cannot substitute for external validation in a separate ACS cohort. Additionally, while the official SYNTAX algorithm requires a ≥ 50% stenosis threshold, visual anatomical assessment at this level may overestimate the functional significance of intermediate lesions. Lastly, the timing of the K10 assessment captures a compound phenotype of pre-existing psychological vulnerability superimposed by an acute, event-triggered stress response.

## 5. Conclusions

In this prospective ACS cohort, K10-defined psychological distress was independently associated with higher SYNTAX I and SYNTAX II PCI scores. K10 showed moderate stand-alone discrimination for higher coronary complexity and improved internally validated prediction when added to clinical variables. These findings support the rationale for integrating brief psychological distress screening into ACS research and potentially into post-ACS care pathways. External validation is required before K10 can be used as a formal risk-stratification component.

## Figures and Tables

**Table 1:** Baseline demographic and clinical characteristics according to K10 distress category.

Variable	Overall (n = 750)	Lower K10 <20 (n = 197)	Mild 20–24 (n = 217)	Moderate 25–29 (n = 203)	Severe ≥30 (n = 133)	*p* Value	*p* for Trend
Age, years	61.3 ± 11.6	60.7 ± 11.2	60.8 ± 11.1	61.2 ± 12.5	62.9 ± 11.6	0.329	0.084
Age ≥ 75 years	105 (14.0%)	24 (12.2%)	28 (12.9%)	29 (14.3%)	24 (18.0%)	0.375	0.104
Male sex	543 (72.4%)	143 (72.6%)	157 (72.4%)	147 (72.4%)	96 (72.2%)	0.984	0.944
Body mass index, kg/m^2^	28.2 ± 4.1	27.8 ± 4.0	28.1 ± 3.9	28.4 ± 4.3	28.7 ± 4.2	0.214	0.049
Diabetes mellitus	205 (27.3%)	45 (22.8%)	58 (26.7%)	55 (27.1%)	47 (35.3%)	0.053	0.014
Hypertension	393 (52.4%)	91 (46.2%)	111 (51.2%)	111 (54.7%)	80 (60.2%)	0.067	0.013
Hyperlipidemia	329 (43.9%)	78 (39.6%)	96 (44.2%)	91 (44.8%)	64 (48.1%)	0.510	0.155
Current smoking	286 (38.1%)	72 (36.5%)	83 (38.2%)	80 (39.4%)	51 (38.3%)	0.953	0.736
Family history of premature CAD	119 (15.9%)	28 (14.2%)	32 (14.7%)	36 (17.7%)	23 (17.3%)	0.721	0.302
Previous myocardial infarction	101 (13.5%)	20 (10.2%)	29 (13.4%)	27 (13.3%)	25 (18.8%)	0.151	0.035
Previous PCI	118 (15.7%)	25 (12.7%)	34 (15.7%)	31 (15.3%)	28 (21.1%)	0.247	0.058
Previous CABG	36 (4.8%)	7 (3.6%)	10 (4.6%)	11 (5.4%)	8 (6.0%)	0.713	0.289
Chronic kidney disease	53 (7.1%)	10 (5.1%)	14 (6.5%)	14 (6.9%)	15 (11.3%)	0.201	0.046
COPD	72 (9.6%)	17 (8.6%)	20 (9.2%)	20 (9.9%)	15 (11.3%)	0.880	0.430
Peripheral vascular disease	43 (5.7%)	8 (4.1%)	12 (5.5%)	12 (5.9%)	11 (8.3%)	0.504	0.118
Prior depression or anxiety diagnosis	82 (10.9%)	11 (5.6%)	20 (9.2%)	26 (12.8%)	25 (18.8%)	<0.001	<0.001
Current antidepressant use	58 (7.7%)	8 (4.1%)	13 (6.0%)	18 (8.9%)	19 (14.3%)	0.002	<0.001
Current anxiolytic use	36 (4.8%)	5 (2.5%)	7 (3.2%)	11 (5.4%)	13 (9.8%)	0.008	0.001
Systolic blood pressure, mmHg	133 ± 24	136 ± 23	134 ± 24	132 ± 25	128 ± 25	0.026	0.006
Heart rate, beats/min	81 ± 16	78 ± 15	80 ± 16	82 ± 16	86 ± 17	<0.001	<0.001
Killip class ≥ 2	139 (18.5%)	27 (13.7%)	36 (16.6%)	42 (20.7%)	34 (25.6%)	0.047	0.006
GRACE risk score	132 ± 31	124 ± 29	130 ± 30	136 ± 31	143 ± 32	<0.001	<0.001
Baseline beta-blocker use	271 (36.1%)	77 (39.1%)	79 (36.4%)	71 (35.0%)	44 (33.1%)	0.681	0.219
Baseline statin use	302 (40.3%)	84 (42.6%)	90 (41.5%)	78 (38.4%)	50 (37.6%)	0.748	0.256
Baseline ACEI/ARB use	336 (44.8%)	88 (44.7%)	97 (44.7%)	91 (44.8%)	60 (45.1%)	0.999	0.967
K10 score	23.7 ± 6.7	15.4 ± 2.9	22.0 ± 1.3	26.9 ± 1.4	33.8 ± 3.4	<0.001	<0.001

Values are mean ± standard deviation or n (%). ACEI/ARB, angiotensin-converting enzyme inhibitor/angiotensin receptor blocker; CABG, coronary artery bypass grafting; CAD, coronary artery disease; COPD, chronic obstructive pulmonary disease; GRACE, Global Registry of Acute Coronary Events; K10, Kessler Psychological Distress Scale-10; PCI, percutaneous coronary intervention.

**Table 2:** Laboratory, echocardiographic, and inflammatory parameters according to K10 distress category.

Variable	Overall (n = 750)	Lower K10 <20	Mild 20–24	Moderate 25–29	Severe ≥30	*p* Value	*p* for Trend
Hemoglobin, g/dL	13.7 ± 1.7	13.9 ± 1.6	13.8 ± 1.7	13.6 ± 1.8	13.4 ± 1.8	0.038	0.006
White blood cell count, 10^3^/uL	10.6 ± 3.2	10.1 ± 2.9	10.5 ± 3.1	10.8 ± 3.3	11.3 ± 3.6	0.012	0.002
Neutrophil count, 10^3^/uL	7.5 ± 2.9	7.0 ± 2.5	7.3 ± 2.7	7.7 ± 3.0	8.3 ± 3.3	0.004	<0.001
Lymphocyte count, 10^3^/uL	2.0 ± 0.8	2.1 ± 0.8	2.0 ± 0.8	1.9 ± 0.7	1.8 ± 0.7	0.029	0.005
Neutrophil-to-lymphocyte ratio	4.2 (2.7–6.4)	3.5 (2.3–5.3)	4.0 (2.6–6.0)	4.5 (2.9–6.8)	5.1 (3.3–7.5)	<0.001	<0.001
Platelet count, 10^3^/uL	244 ± 69	239 ± 66	242 ± 67	246 ± 70	252 ± 73	0.331	0.085
Mean platelet volume, fL	9.7 ± 1.1	9.5 ± 1.0	9.6 ± 1.1	9.8 ± 1.1	10.0 ± 1.2	0.006	<0.001
Creatinine, mg/dL	1.02 ± 0.34	0.98 ± 0.29	1.01 ± 0.32	1.03 ± 0.35	1.10 ± 0.42	0.015	0.003
Creatinine clearance, mL/min	58.3 ± 17.7	59.6 ± 16.9	58.8 ± 17.5	57.9 ± 18.2	56.7 ± 18.1	0.541	0.118
Fasting glucose, mg/dL	137 ± 58	128 ± 49	134 ± 55	139 ± 59	151 ± 70	0.004	<0.001
HbA1c, %	6.7 ± 1.5	6.4 ± 1.3	6.6 ± 1.4	6.8 ± 1.5	7.1 ± 1.7	0.002	<0.001
LDL-C, mg/dL	124.2 ± 32.9	123.9 ± 31.3	124.6 ± 33.4	125.0 ± 34.4	122.8 ± 32.2	0.940	0.901
HDL-C, mg/dL	41.5 ± 10.2	42.7 ± 10.5	41.8 ± 10.1	40.9 ± 9.8	39.7 ± 10.4	0.047	0.008
Triglycerides, mg/dL	166 (118–222)	158 (112–210)	163 (116–219)	169 (121–228)	178 (128–239)	0.101	0.031
Peak troponin I, ng/L	4313 (1180–7930)	3510 (920–6620)	4150 (1140–7590)	4620 (1300–8360)	5280 (1540–9260)	0.018	0.004
hs-CRP, mg/L	4.4 ± 3.0	3.6 ± 3.0	4.2 ± 2.6	4.8 ± 3.1	5.6 ± 2.9	<0.001	<0.001
NT-proBNP, pg/mL	810 (290–1960)	610 (210–1450)	740 (260–1760)	880 (330–2140)	1150 (420–2760)	0.002	<0.001
LVEF, %	49.5 ± 7.3	49.2 ± 6.7	49.5 ± 7.6	49.9 ± 7.5	49.6 ± 7.3	0.860	0.682
LVEF <40%	92 (12.3%)	20 (10.2%)	25 (11.5%)	26 (12.8%)	21 (15.8%)	0.481	0.130

Values are mean ± standard deviation, median (interquartile range), or n (%). HDL-C, high-density lipoprotein cholesterol; hs-CRP, high-sensitivity C-reactive protein; LDL-C, low-density lipoprotein cholesterol; LVEF, left ventricular ejection fraction; NT-proBNP, N-terminal pro-B-type natriuretic peptide.

**Table 3:** ACS presentation, early management, and in-hospital course according to K10 distress category.

Variable	Overall (n = 750)	Lower K10 <20	Mild 20–24	Moderate 25–29	Severe ≥30	*p* Value	*p* for Trend
STEMI	337 (44.9%)	83 (42.1%)	89 (41.0%)	101 (49.8%)	64 (48.1%)	0.221	0.096
NSTEMI	310 (41.3%)	78 (39.6%)	102 (47.0%)	72 (35.5%)	58 (43.6%)	0.138	0.838
USAP	103 (13.7%)	36 (18.3%)	26 (12.0%)	30 (14.8%)	11 (8.3%)	0.078	0.038
Symptom-to-door time, hours	5.8 (3.1–11.6)	4.9 (2.7–9.7)	5.5 (3.0–10.9)	6.1 (3.2–12.4)	7.4 (3.8–15.1)	0.006	<0.001
Anterior STEMI among STEMI	142/337 (42.1%)	32/83 (38.6%)	36/89 (40.4%)	45/101 (44.6%)	29/64 (45.3%)	0.686	0.247
Door-to-balloon time in STEMI, min	58 (45–74)	56 (43–72)	57 (44–75)	60 (47–75)	61 (48–77)	0.346	0.103
Radial access	616 (82.1%)	166 (84.3%)	181 (83.4%)	164 (80.8%)	105 (78.9%)	0.542	0.189
Primary PCI for STEMI	318/337 (94.4%)	78/83 (94.0%)	85/89 (95.5%)	95/101 (94.1%)	60/64 (93.8%)	0.956	0.795
Aspirin within first 24 h	746 (99.5%)	196 (99.5%)	216 (99.5%)	202 (99.5%)	132 (99.2%)	0.987	0.757
P2Y12 inhibitor within first 24 h	739 (98.5%)	194 (98.5%)	214 (98.6%)	200 (98.5%)	131 (98.5%)	0.999	0.980
High-intensity statin during hospitalization	721 (96.1%)	191 (97.0%)	210 (96.8%)	194 (95.6%)	126 (94.7%)	0.687	0.220
Glycoprotein IIb/IIIa inhibitor	104 (13.9%)	21 (10.7%)	27 (12.4%)	31 (15.3%)	25 (18.8%)	0.202	0.029
Inotropic/vasopressor support	58 (7.7%)	10 (5.1%)	14 (6.5%)	18 (8.9%)	16 (12.0%)	0.141	0.017
Mechanical circulatory support	17 (2.3%)	3 (1.5%)	4 (1.8%)	5 (2.5%)	5 (3.8%)	0.591	0.174
New-onset atrial fibrillation	31 (4.1%)	5 (2.5%)	8 (3.7%)	9 (4.4%)	9 (6.8%)	0.337	0.062
In-hospital heart failure	76 (10.1%)	13 (6.6%)	20 (9.2%)	23 (11.3%)	20 (15.0%)	0.092	0.011
Major bleeding	21 (2.8%)	4 (2.0%)	6 (2.8%)	6 (3.0%)	5 (3.8%)	0.846	0.389
Length of stay, days	4.2 ± 2.1	3.8 ± 1.8	4.0 ± 2.0	4.4 ± 2.2	4.8 ± 2.4	<0.001	<0.001

Values are mean ± standard deviation, median (interquartile range), or n (%). ACS, acute coronary syndrome; NSTEMI, non-ST-segment elevation myocardial infarction; PCI, percutaneous coronary intervention; STEMI, ST-segment elevation myocardial infarction; USAP, unstable angina pectoris.

**Table 4:** Angiographic, lesion, and procedural characteristics according to K10 distress category.

Variable	Overall (n = 750)	Lower K10 <20	Mild 20–24	Moderate 25–29	Severe ≥30	*p* Value	*p* for Trend
Culprit left anterior descending artery	302 (40.3%)	70 (35.5%)	84 (38.7%)	88 (43.3%)	60 (45.1%)	0.211	0.043
Culprit circumflex artery	151 (20.1%)	42 (21.3%)	45 (20.7%)	39 (19.2%)	25 (18.8%)	0.913	0.504
Culprit right coronary artery	238 (31.7%)	65 (33.0%)	70 (32.3%)	62 (30.5%)	41 (30.8%)	0.949	0.610
Left main culprit/major lesion	40 (5.3%)	9 (4.6%)	11 (5.1%)	13 (6.4%)	7 (5.3%)	0.868	0.617
Multivessel disease	365 (48.7%)	70 (35.5%)	93 (42.9%)	111 (54.7%)	91 (68.4%)	<0.001	<0.001
Three-vessel disease	184 (24.5%)	29 (14.7%)	44 (20.3%)	56 (27.6%)	55 (41.4%)	<0.001	<0.001
Chronic total occlusion	91 (12.1%)	14 (7.1%)	23 (10.6%)	29 (14.3%)	25 (18.8%)	0.008	<0.001
Bifurcation lesion	168 (22.4%)	31 (15.7%)	46 (21.2%)	50 (24.6%)	41 (30.8%)	0.018	0.002
Severe calcification	143 (19.1%)	25 (12.7%)	36 (16.6%)	42 (20.7%)	40 (30.1%)	<0.001	<0.001
Visible thrombus	236 (31.5%)	54 (27.4%)	65 (30.0%)	67 (33.0%)	50 (37.6%)	0.264	0.054
Long lesion >20 mm	287 (38.3%)	57 (28.9%)	78 (35.9%)	85 (41.9%)	67 (50.4%)	<0.001	<0.001
Ostial/proximal lesion	154 (20.5%)	30 (15.2%)	42 (19.4%)	45 (22.2%)	37 (27.8%)	0.047	0.005
Number of implanted stents	1.42 ± 0.84	1.18 ± 0.71	1.35 ± 0.79	1.50 ± 0.85	1.78 ± 0.94	<0.001	<0.001
Total stent length, mm	31 (18–48)	24 (15–38)	29 (17–45)	34 (20–51)	40 (24–60)	<0.001	<0.001
Contrast volume, mL	142 ± 55	126 ± 47	137 ± 51	149 ± 56	166 ± 63	<0.001	<0.001
Procedural success	711 (94.8%)	191 (97.0%)	207 (95.4%)	190 (93.6%)	123 (92.5%)	0.236	0.044
Complete revascularization	392 (52.3%)	121 (61.4%)	117 (53.9%)	99 (48.8%)	55 (41.4%)	0.002	<0.001
SYNTAX I score	22.4 ± 9.5	17.2 ± 8.7	21.8 ± 8.5	24.3 ± 8.8	28.4 ± 8.5	<0.001	<0.001
Low SYNTAX I <=22	363 (48.4%)	147 (74.6%)	123 (56.7%)	90 (44.3%)	33 (24.8%)	<0.001	<0.001
Intermediate SYNTAX I 23–32	266 (35.5%)	38 (19.3%)	73 (33.6%)	85 (41.9%)	70 (52.6%)	<0.001	<0.001
High SYNTAX I ≥33	121 (16.1%)	12 (6.1%)	21 (9.7%)	28 (13.8%)	30 (22.6%)	<0.001	<0.001
SYNTAX I ≥23	387 (51.6%)	50 (25.4%)	94 (43.3%)	113 (55.7%)	100 (75.2%)	<0.001	<0.001
SYNTAX II PCI score	31.2 ± 9.6	27.0 ± 8.2	30.2 ± 9.0	32.7 ± 9.6	36.7 ± 9.4	<0.001	<0.001
SYNTAX II CABG score	29.8 ± 8.9	26.1 ± 7.8	29.0 ± 8.2	31.2 ± 9.0	34.4 ± 8.9	<0.001	<0.001
SYNTAX II PCI ≥36	220 (29.3%)	31 (15.7%)	53 (24.4%)	67 (33.0%)	69 (51.9%)	<0.001	<0.001

Values are mean ± standard deviation, median (interquartile range), or n (%). CABG, coronary artery bypass grafting; PCI, percutaneous coronary intervention. The lesion-level variables are shown to make the anatomical source of the higher SYNTAX burden transparent.

## Data Availability

The data presented in this study are available on request from the corresponding author. The data are not publicly available due to privacy and ethical restrictions.

## References

[1] Byrne R.A., Rossello X., Coughlan J.J., Barbato E., Berry C., Chieffo A., Claeys M.J., Dan G.-A., Dweck M.R., Galbraith M. (2023). 2023 ESC Guidelines for the management of acute coronary syndromes: Developed by the task force on the management of acute coronary syndromes of the European Society of Cardiology (ESC). Eur. Heart J..

[2] Kozan O., Ergene O., Oto A., Kaplan A.K., TURK-AKS Investigators (2017). A real life registry to evaluate patient profile, diagnostic and practice patterns in acute coronary syndrome in Turkey: TURK-AKS study. Int. J. Cardiovasc. Acad..

[3] Erol M.K., Kayikcioglu M., Kilickap M., Arın C.B., Kurt İ H., Aktaş İ., Güneş B.Y., Özkan E., Şen T.Z., Ince O. (2020). Baseline clinical characteristics and patient profile of the TURKMI registry: Results of a nation-wide acute myocardial infarction registry in Turkey. Anatol. J. Cardiol..

[4] Sianos G., Morel M.A., Kappetein A.P., Morice M.C., Colombo A., Dawkins K., Brand M.V.D., Van Dyck N., E Russell M., Mohr F.W. (2005). The SYNTAX Score: An angiographic tool grading the complexity of coronary artery disease. EuroIntervention.

[5] Serruys P.W., Onuma Y., Garg S., Sarno G., van den Brand M., Kappetein A.P., Van Dyck N., Mack M., Holmes D., Feldman T. (2009). Assessment of the SYNTAX score in the SYNTAX study. EuroIntervention.

[6] Palmerini T., Genereux P., Caixeta A., Cristea E., Lansky A., Mehran R., Dangas G., Lazar D., Sanchez R., Fahy M. (2011). Prognostic value of the SYNTAX score in patients with acute coronary syndromes undergoing percutaneous coronary intervention: Analysis from the ACUITY trial. J. Am. Coll. Cardiol..

[7] Farooq V., van Klaveren D., Steyerberg E.W., Meliga E., Vergouwe Y., Chieffo A., Kappetein A.P., Colombo A., Holmes D.R., Mack M. (2013). Anatomical and clinical characteristics to guide decision making between coronary artery bypass surgery and percutaneous coronary intervention for individual patients: Development and validation of SYNTAX score II. Lancet.

[8] Kessler R.C., Andrews G., Colpe L.J., Hiripi E., Mroczek D.K., Normand S.L.T., Walters E.E., Zaslavsky A.M. (2002). Short screening scales to monitor population prevalences and trends in non-specific psychological distress. Psychol. Med..

[9] Stolk Y., Kaplan I., Szwarc J. (2014). Clinical use of the Kessler psychological distress scales with culturally diverse groups. Int. J. Methods Psychiatr. Res..

[10] Thakre M.N., Sathe H.S., Talapalliwar M.R. (2022). Psychometric properties of Kessler’s Psychological Distress Scale (K10) in cancer patients. Arch. Ment. Health.

[11] Andrews G., Slade T. (2001). Interpreting scores on the Kessler Psychological Distress Scale (K10). Aust. N. Z. J. Public Health.

[12] Altun Y., Ozen M., Kuloglu M.M. (2019). Turkish adaptation of Kessler Psychological Distress Scale: Validity and reliability study. Anadolu Psikiyatr. Derg..

[13] Welsh J., Banks E., Joshy G., Butterworth P., Strazdins L., Korda R.J. (2021). Does psychological distress directly increase risk of incident cardiovascular disease? Evidence from a prospective cohort study using a longer-term measure of distress. BMJ Open.

[14] Jackson C.A., Sudlow C.L.M., Mishra G.D. (2018). Psychological distress and risk of myocardial infarction and stroke in the 45 and Up Study: A prospective cohort study. Circ. Cardiovasc. Qual. Outcomes.

[15] Pimple P., Lima B.B., Hammadah M., Wilmot K., Ramadan R., Levantsevych O., Sullivan S., Kim J.H., Kaseer B., Shah A.J. (2019). Psychological distress and subsequent cardiovascular events in individuals with coronary artery disease. J. Am. Heart Assoc..

[16] Xiao Y., Zhang S., Ma X., Yang Y., Tan X., Li J. (2019). Impact of depression and/or anxiety on patients with acute coronary syndrome after percutaneous coronary intervention: A systematic review and meta-analysis. BMJ Open.

[17] Carney R.M., Freedland K.E. (2017). Depression and coronary heart disease. Nat. Rev. Cardiol..

[18] Whooley M.A., Wong J.M. (2013). Depression and cardiovascular disorders. Annu. Rev. Clin. Psychol..

[19] Rozanski A., Blumenthal J.A., Davidson K.W., Saab P.G., Kubzansky L. (2005). The epidemiology, pathophysiology, and management of psychosocial risk factors in cardiac practice. J. Am. Coll. Cardiol..

[20] Steptoe A., Kivimaki M. (2012). Stress and cardiovascular disease. Nat. Rev. Cardiol..

[21] Huffman J.C., Celano C.M., Beach S.R., Motiwala S.R., Januzzi J.L. (2013). Depression and cardiac disease: Epidemiology, mechanisms, and diagnosis. Cardiovasc. Psychiatry Neurol..

[22] Kaya A.F., Yilmaz C., Ozdil M.H., Soner S., Ozbek M. (2024). Is there a relationship between anxiety-depression level and SYNTAX score in patients with acute coronary syndrome undergoing percutaneous coronary intervention?. Kosuyolu Heart J..

[23] Ekici B., Ercan E.A., Cehreli S., Töre H.F. (2014). The effect of emotional status and health-related quality of life on the severity of coronary artery disease. Kardiol. Pol..

[24] von Elm E., Altman D.G., Egger M., Pocock S.J., Gøtzsche P.C., Vandenbroucke J.P., STROBE Initiative (2007). The strengthening the reporting of observational studies in epidemiology (STROBE) statement: Guidelines for reporting observational studies. Ann. Intern. Med..

[25] Granger C.B., Goldberg R.J., Dabbous O., Pieper K.S., Eagle K.A., Cannon C.P., Van De Werf F., Avezum A., Goodman S.G., Flather M.D. (2003). Predictors of hospital mortality in the Global Registry of Acute Coronary Events. Arch. Intern. Med..

[26] Fox K.A.A., Fitzgerald G., Puymirat E., Huang W., Carruthers K., Simon T., Coste P., Monsegu J., Steg P.G., Danchin N. (2014). Should patients with acute coronary disease be stratified for management according to their risk? Derivation, external validation and outcomes using the updated GRACE risk score. BMJ Open.

